# The effects of anthropogenic and volcanic aerosols and greenhouse gases on twentieth century Sahel precipitation

**DOI:** 10.1038/s41598-020-68356-w

**Published:** 2020-07-22

**Authors:** Rebecca Jean Herman, Alessandra Giannini, Michela Biasutti, Yochanan Kushnir

**Affiliations:** 1 Department of Earth and Environmental Sciences, Columbia University, New York, NY USA; 2 International Research Institute for Climate and Society, The Earth Institute at Columbia University, New York, NY USA; 3 Laboratoire de Météorologie Dynamique/IPSL, École Normale Supérieure, PSL Research University, Sorbonne Université, École Polytechnique, IP Paris, CNRS, Paris, France; 4 Lamont-Doherty Earth Observatory of Columbia University, Palisades, NY USA

**Keywords:** Atmospheric dynamics, Attribution

## Abstract

There is little scientific consensus on the importance of external climate forcings—including anthropogenic aerosols, volcanic aerosols, and greenhouse gases (GHG)—relative to each other and to internal variability in dictating past and future Sahel rainfall. We address this query by relating a 3-tiered multi-model mean (MMM) over the Climate Model Intercomparison Project phase 5 “twentieth century” and pre-Industrial control simulations to observations. The comparison of single-forcing and historical simulations highlights the importance of anthropogenic and volcanic aerosols over GHG in generating forced Sahel rainfall variability in models. However, the forced MMM only accounts for a small fraction of observed variance. A residual consistency test shows that simulated internal variability cannot explain the residual observed multidecadal variability, and points to model deficiency in simulating multidecadal variability in the forced response, internal variability, or both.

## Introduction

The Sahel—the boundary between the North African Savanna and the Sahara Desert—experienced dramatic, long-term rainfall variability in the twentieth century which was unparalleled in the rest of the world. This variability was marked by a striking decline in rainfall between about 1960 and the early 1980s, including devastating droughts and famine in the early 1970s and 80s, which left 100,000 people dead and 750,000 dependent on food aid^1^. Scientific work immediately began to explore potential relationships between Sahel rainfall and a wide variety of local^2,3^ and global^4,5^ climatic factors. Giannini, et al.^6^ confirmed the importance of global over local processes by showing that an atmospheric model forced with observed global sea surface temperature (SST) alone could reproduce the profile of the first principal component of Sahel twentieth century rainfall variability, if not the amplitude, at a correlation of approximately 0.7. Studies since then have continued to focus on various global processes, reinforcing the connections between the Sahel and the temperature of ocean basins across the globe, and establishing links to internal variability—such as the El Niño-Southern Oscillation (ENSO)^7–9^ and the Atlantic Multidecadal Oscillation (AMO)^9,10^—and external forcing—such as greenhouse gases (GHG)^11–16^ and volcanic and anthropogenic aerosols^13,17,18^. However, the relative importance of internal variability and different sources of external forcing remain unclear.

There is a developing consensus in the literature that anthropogenic aerosols have contributed to the Sahel drought, though there is disagreement over the prominence of this contribution and the physical mechanism that governs it^10,11,13,16–26^. The magnitude of the contribution is somewhat contentious because of disagreement about the strength of the indirect aerosol effects^27–29^, which may influence SSTs and global precipitation much more than the direct radiative effect^30–32^, and which may cause non-linear interactions affecting both the spatial pattern (i.e. Polson et al.^18^ on the Asian monsoon) and even the mean^33^ of the precipitation and temperature responses to other sources of forcing. The role of greenhouse gases (GHG) is even more widely debated—not just in the twentieth century^12–17,22,26,30^, but even in the future when GHG forcing dominates^11,12,15,19^. Some argue that there are also non-linear interactions between different effects of increasing GHG^34,35^ or between GHG and other external forcings^16^ and internal processes^36^. Finally, many studies claim that SST and Sahel rainfall variation are primarily of internal origin^37–39^.

Many of the above studies on the Sahel focus on one or two types of forcing or on one model, or are limited to CMIP3^40^, in which most models did not include indirect aerosol effects. Some, such as Giannini and Kaplan^16^, use a storyline approach—focusing on proposing physically-consistent pathways in order to avoid underestimating regional impacts^41^. Others^18,23^ use fingerprinting, extracting distinct spatial and/or temporal patterns associated with different forcings and scaling the model response to match observations in order to correct sensitivity biases and avoid compensating errors in the models^42^.

We attempt to enrich the debate about the influence of external forcing and internal variability on Sahel rainfall over the twentieth century by performing an attribution study using the Coupled Model Intercomparison Project phase 5 (CMIP5)^43^, which is the first large ensemble of coupled models to include aerosol indirect effects and run “single-forcing” model simulations, in which one external source of radiative forcing—such as greenhouse gases (GHG), anthropogenic aerosols (AA), or natural forcing (which includes volcanic aerosols and solar and orbital variations, NAT)—varies historically while the other external forcings are held at constant pre-Industrial values. We compare the evolution of spatially- and seasonally-averaged July–September (JAS) observed Sahel rainfall to that of the twentieth century single-forcing and “historical” simulations, in which all external forcings vary simultaneously (ALL). We determine the forced responses via a weighted, tiered multi-model mean (MMM) of CMIP5 simulations (see “Methods” for details), and then calculate correlation coefficients and root mean squared errors (RMSE, expressed as fraction of observed variance) to estimate the contributions of different external forcings to observed precipitation variability, using bootstrapping methods to estimate uncertainty in those statistics. To estimate noise in the MMM and significance, we use the long preindustrial control (piC) simulations, in which all external radiative forcings are held at constant preindustrial values. We employ spectral analysis of individual twentieth century and piC simulations to estimate the contribution of internal variability to observed precipitation variability at multidecadal time scales.

## Results

### Multi-model mean performance

For each forcing experiment, we compute the MMM as follows: (1) an average across individual runs gives the ensemble mean (EM) for each CMIP5 model, (2) a weighted average across EMs gives the institution mean (IM) for each participating research institution, (3) and a weighted average across IMs gives the multi-model mean (MMM). The weights are designed to counteract attenuation of noise in ensemble and institution means that include more runs and EMs, respectively, so that they will not be underrepresented in the MMM relative to their noisier and more variable counterparts. For a formula and its derivation, see “The multi-model mean” under “Methods”.

**Figure 1:**
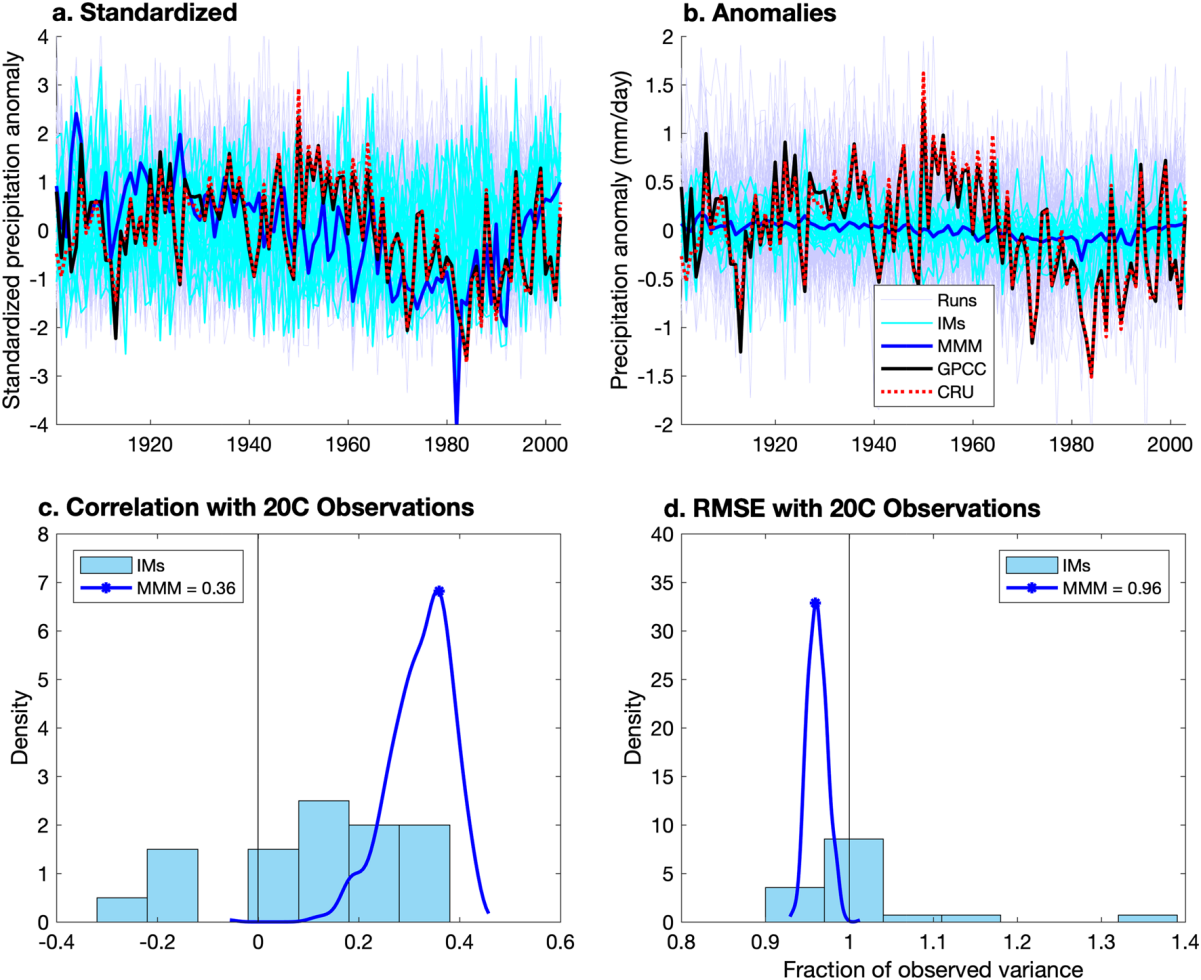
MMM Performance: Standardized (**a**) and actual (**b**) departures from climatology of twentieth century Sahel precipitation in individual ALL runs (blue–grey solid lines), ALL institution means (IMs, cyan), the ALL MMM (blue), and observations from GPCC (black) and CRU (red dotted line). Histogram (cyan) of correlations (**c**) and RMSE (**d**) between GPCC observations and the IMs, actual correlation (**c**) and RMSE (**d**) of the MMM with GPCC observations (blue dot), and the bootstrapping PDFs (blue curve) of the correlation (**c**) and RMSE (**d**) between the ALL MMM and GPCC observations.

In Fig. 1a,b, we compare Sahel twentieth century precipitation anomalies for the ALL MMM (blue line) to individual ALL runs (blue-grey lines, background) and IMs (cyan lines), and to observations from the Global Precipitation Climatology Center^44^ (GPCC, black line) and the Climatic Research Unit^45^ (CRU, red dotted line). Despite disagreement in the first three years, the spatial averages of the two observational records look similar enough that uncertainty in area-averaged Sahelian precipitation is considered small, and only GPCC is used throughout the rest of the paper. Despite the spread of the IMs, the standardized anomalies (Fig. 1a) reveal a striking similarity between observations and the MMM, which captures much of the observations’ multi-decadal variation by reproducing the drought of the 70s and 80s and its recovery, and even many episodes of dramatic interannual rainfall changes, most notably near 1984, the driest year in observations. Assuming the averaging was successful in preferentially filtering out internal variability present in individual model simulations, the MMM represents a consensus, forced Sahelian rainfall profile which is recognizable in the observations (Fig. 1a). However, the actual rainfall anomalies (Fig. 1b) reveal substantial attenuation of variance in the ALL MMM compared to individual simulations and to the observations.

The remaining panels of Fig. 1 display the correlations (Fig. 1c) and the RMSE (Fig. 1d) of individual IMs (cyan histogram) and of the MMM (blue dot) with observations. The blue curves show probability density functions (PDF) from bootstrapping over the IMs (see “Methods”), and represent how those statistics might change with a slightly different set of models. The correlation measures the similarity in the shape of one time series with respect to the other but is independent of relative amplitude, whereas the RMSE estimates the difference in amplitude of the simulated and observed yearly rainfall time series.

The MMM performs as well as or better than most individual IMs in both metrics, consistent with previous studies which compared other versions of multi-model means to individual models^46^. Though some research institutions may appear to outperform the MMM in correlation and RMSE with twentieth century observations (notably, GISS outperforms the MMM in both), as we are comparing only one variable (precipitation) to one realization of observations in which forced and internal variability are indistinguishable, it is unclear whether these models truly capture the underlying mechanisms better than the ensemble. The RMSE values for the MMM and the IMs are near 100% of observed variance, partially reflecting the severe attenuation seen in Fig. 1b.

### Model response to different forcing experiments

**Figure 2:**
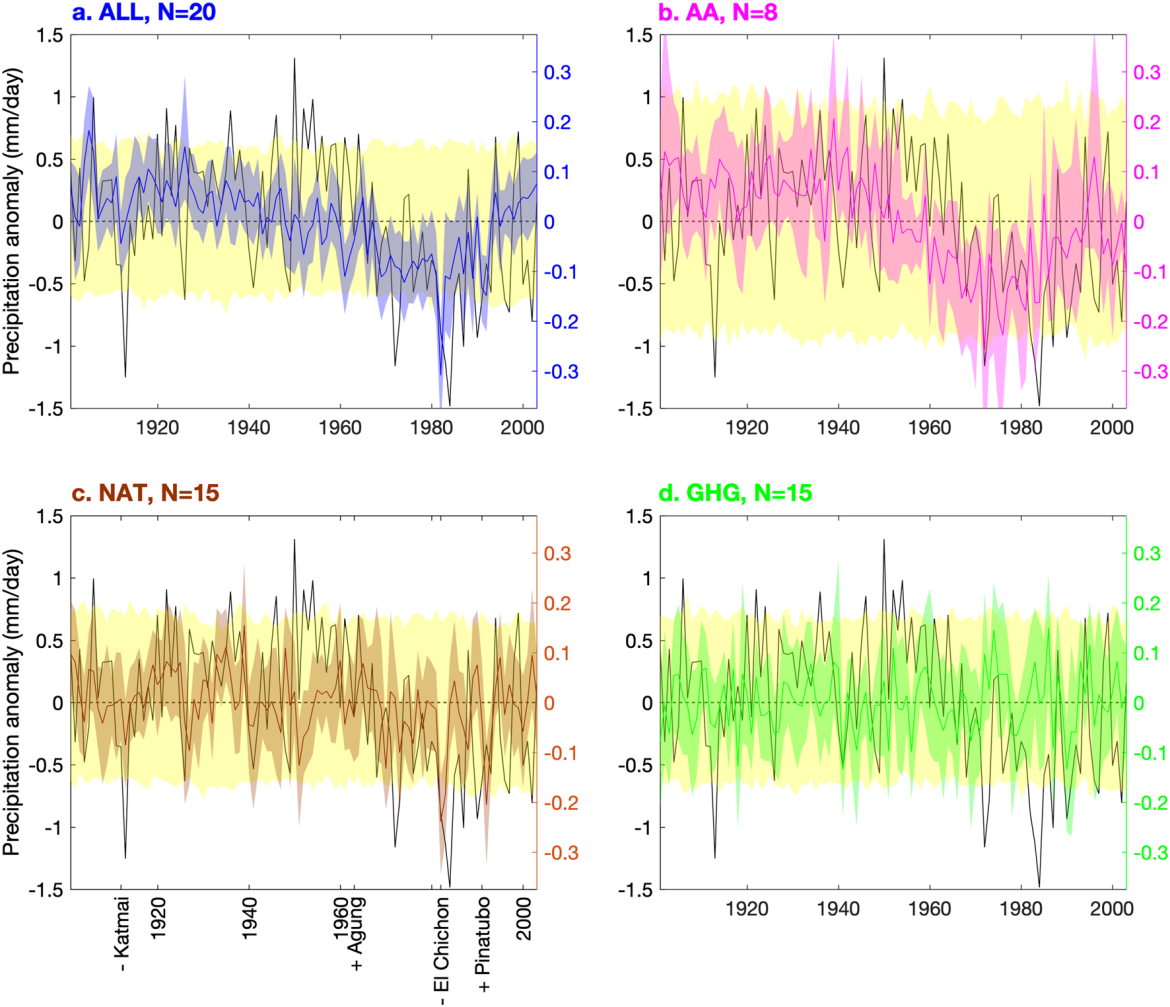
Forced MMMs: Forced MMM Sahel precipitation anomalies (colored lines; right, colored ordinates) and their yearly 95% confidence intervals from bootstrapping (colored shaded areas; right, colored ordinates) over observed Sahel precipitation anomalies (black lines; left, black ordinates) and the 95% confidence interval of the piC runs from randomized bootstrapping (yellow shaded areas; right, colored ordinates). N are the number of research institutions which performed each forcing experiment. Panel (c) additionally identifies the dates of large volcanic eruptions which had different effects on the aerosol optical depth in the northern and the southern hemispheres, as well as the sign of that difference^17^.

Figure 2 displays the MMMs for the three different single-forcing experiments: AA for **a**nthropogenic **a**erosols (pink, Fig. 2b), NAT for **nat**ural forcing (brown, Fig. 2c), and GHG for **g**reen**h**ouse **g**ases (green, Fig. 2d); and compares them to observations (black). Figure 2a again displays the ALL MMM (blue). Note that the observations correspond to the black ordinates on the left, while forced and piC model outputs (colors, including yellow) correspond to the colored ordinates on the right, which have a scale a quarter the range to facilitate comparison. The blue, pink, brown, and green shaded areas are the 95% range of bootstrapped forced MMMs. They represent agreement in the forced signal between the institutions, even though, due to small sample size, they do not fully capture the magnitude of noise in the MMM caused by coincident simulated internal variability (see “Uncertainty and significance: bootstrapping and randomized bootstrapping” under “Methods”). The yellow shaded areas are also a 95% confidence interval, but they are obtained using randomly-chosen continuous subsequences of the piC runs in place of the historical simulations, where the piC simulations are taken from the same set of research institutions which provided simulations for that historical forcing experiment. The yellow shading captures the magnitude of noise in the MMM; however, it is worth note that it may be a slight overestimate since the MMM is less effective at filtering noise when there are fewer runs per model, and there is almost always only one piC run per model.

The variance of the forced MMMs over time (solid lines) and of the bootstrapped forced MMMs and randomized bootstrapped piC MMMs in a given year (shaded areas) vary from panel to panel inversely (though not proportionally) with the square root of the number of research institutions which simulated each forcing experiment (N), and are all roughly a quarter of observed variance, consistent with many precipitation fingerprinting studies, which often scale simulated precipitation up by a factor of 3–5^18,23,42,47^. Aside from a few exceptions, the yearly magnitudes of the forced MMMs are not significant, as they do not surpass the yellow zone consistent with noise in the MMM; this limits the detail with which we can examine the MMM directly. However, NAT (Fig. 2c) and ALL (Fig. 2a) are both significantly dry in 1982 (the year of the El Chichón eruption, near the driest observed year in 1984), and AA (Fig. 2b) and ALL both display multi-decadal variability in the second half of the century (including a partial recovery) which is characteristic of the observations and uncharacteristic of NAT and GHG (Fig. 2d).

**Figure 3:**
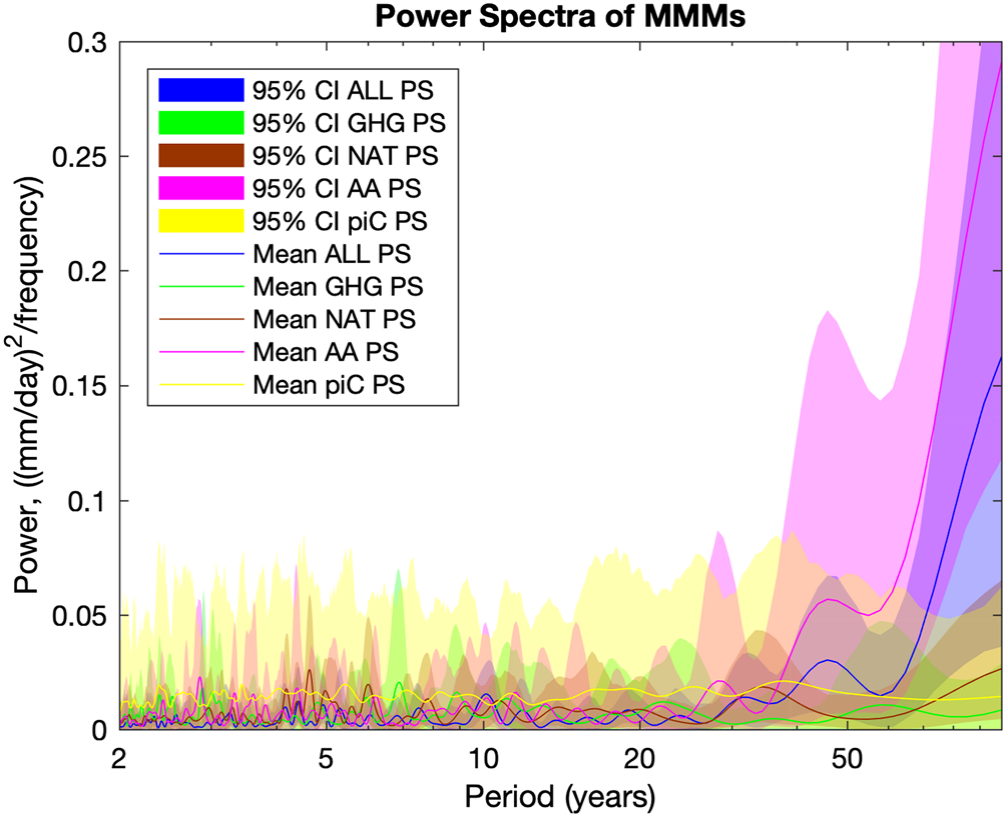
Forced MMM power spectra: mean (lines) and 95% confidence intervals (shaded areas) of padded power spectra (PS) of bootstrapped forced MMMs (ALL—blue, NAT—brown, AA—pink, GHG—green) and randomized bootstrapped AA piC MMMs (yellow).

Figure 3 displays the mean padded power spectra (PS, lines) and 95% confidence intervals (shaded areas) of the bootstrapped forced MMMs (colors other than yellow), and compares them to that of the randomized bootstrapped piC MMMs (yellow). We calculate the piC MMM using the reduced set of models that contributed the AA experiment. With only 8 contributing research institutions, the AA MMMs filter out less noise from modelled internal variability—and thus have more power at all frequencies—than the MMMs associated with the other experiments. Thus, using this reduced set of models provides a conservative estimate of the spectral noise in all four forcing experiments. Figure 3 shows that the multi-decadal variability in AA (pink) and ALL (blue) is distinct from noise (yellow). It also confirms that the high-frequency variability in GHG is consistent with noise. Episodic volcanic forcing should not give rise, per se, to spectral peaks, though the observed pattern of large eruptions at the beginning and at the end of the century (see Fig. 2c) may induce some spectral power at multidecadal timescales. Since we do not detect any meaningful spectral peak in the NAT PS (brown) associated with solar variability at 11 years, we interpret the NAT MMM to be mostly the result of volcanic aerosols.

**Figure 4:**
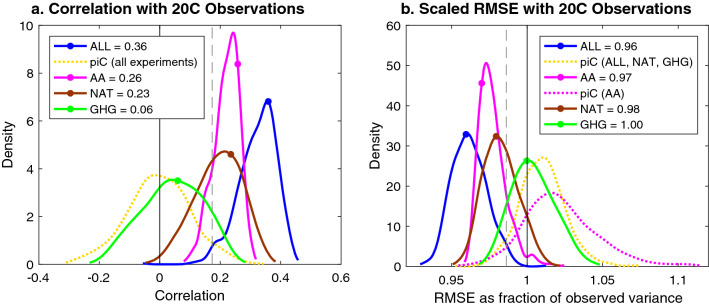
Performance of forced MMMs: probability density function (PDF) of correlations (**a**) and RMSE (**b**) of bootstrapped forced MMM twentieth century Sahel precipitation (colored curves: blue = ALL, pink = AA, brown = NAT, green = GHG) and of randomized bootstrapped piC MMM Sahel precipitation corresponding to the ALL experiment (dotted yellow curves) and the AA experiment (dotted pink curve, **b**) with observed twentieth century Sahel precipitation. Actual forced MMM values are represented with colored dots on the PDFs. One-sided 95% confidence level represented with grey vertical dashed lines.

Figure 4 again displays the values (dots) and PDFs (curves) of correlation (Fig. 4a) and RMSE (Fig. 4b) between observations and the bootstrapped ALL MMMs from Fig. 1c, d (blue) and compares them to the values (dots) and PDFs (curves) for individual forcing experiments (solid curves distinguished by color) and the piC PDFs associated with the ALL experiment (dotted yellow curves). The piC PDFs corresponding to the three individual forcing experiments (which make use of only the models contributing to that experiment) are sufficiently similar to the ALL piC PDF that they are not plotted separately, with the exception of the AA piC RMSE PDF (pink dotted curve in Fig. 4b), which is wider and centered at a higher RMSE than those of the other experiments, reflecting the high variance in the yearly values seen in the yellow shaded area in Fig. 2b. Despite this difference, the *p* = 0.05 significance levels are still sufficiently similar for all four experiments for both correlation and RMSE that they are represented by a single vertical grey dashed line at the *p* = 0.05 significance level of the ALL experiment. As the NAT and GHG MMMs contain mostly high-frequency variability—which is difficult to distinguish from noise remaining in the MMM (see Fig. 3)—their PDFs are wider than the PDFs for the AA and ALL MMMs, which exhibit low-frequency variability uncharacteristic of noise in the MMM.

While the GHG MMM is not significantly better than noise at matching observations in both correlation (r = 0.06) and RMSE (100% of observed variance), ALL (r = 0.36, RMSE = 0.96), AA (r = 0.26, RMSE = 0.97), and NAT (r = 0.23, RMSE = 0.98) all achieve significance at *p* = 0.05. The discrepancy between the ALL MMM and NAT and AA individually under both metrics suggests that both anthropogenic and volcanic aerosols contribute substantially to the performance of the ALL MMM. Because the metrics for both AA and NAT fall within the other’s bootstrapping confidence interval, according to this analysis, AA and NAT contribute roughly equally to the performance of the observed ALL MMM.

The ALL MMM has limited predictive power as it is nearly constant, and, according to the RMSE, it leaves 96% of the variance unexplained. This unexplained variance could be due either to model deficiency or internal variability, since the MMM is designed to filter out internal variability—which will have similar characteristics but different phase across individual simulations—in favor of forced variability. Since observations include both internal and forced variability, no MMM would be able to match observations exactly. In this light, the ALL MMM correlation with observations of 0.36 is substantial. For comparison, we may liken this to simulations forced with observed SST, which reflect as best as possible observed internal climate variability as well as forced variability. As reported in Giannini et al.^6^, the correlation of the unsmoothed observations with the unsmoothed mean over version 1 of the atmospheric general circulation model developed at NASA’s Goddard Space Flight Center in the framework of the Seasonal-to-Interannual Prediction Project (NSIPP1) from 1930–2000 is 0.60; the correlation of the ALL MMM with observations over the same period is not far behind at 0.47, suggesting that a large fraction of the variability that SST-forced climate models can capture is externally forced.

### Residual consistency

**Figure 5:**
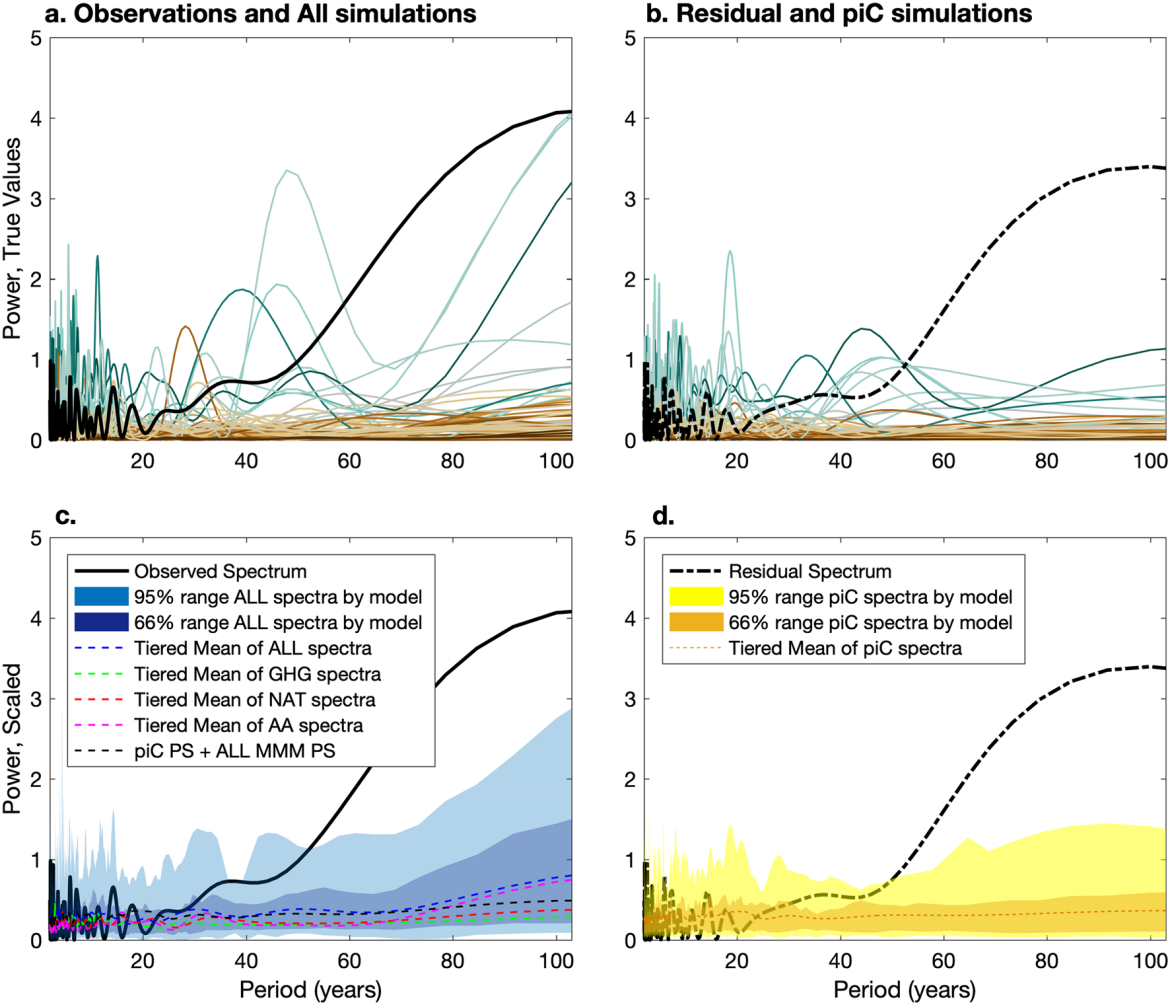
Residual consistency: power spectra (PS) of observed twentieth century Sahel rainfall (solid black, **a** and **c**) and the residual after removing the ALL MMM (black dotted-dashed, **b** and **d**). (**a**) and (**b**): Mean PS by model of individual ALL (**a**) and piC (**b**) runs, colored by average JAS rainfall bias of the ALL runs compared to twentieth century observations, where observed rainfall is grey, wet models are turquoise, and dry models are brown. piC PS (**b**) are additionally averaged over multiple subsections of the runs. (**c**) Tiered mean (blue dashed line) and 66% and 95% range (blue shading) of mean PS by model of individual ALL runs which were first rescaled to match twentieth century observed JAS rainfall. Also displayed are the tiered means over PS of individual forced AA, NAT, and GHG runs (colored dashed lines). The black dashed line shows the sum of the tiered mean piC PS (from panel **d**) and the ALL MMM PS (i.e. Fig. 3). (**d**): Tiered mean (orange dashed line) and 66% and 95% range (yellow shading) of mean PS by model of individual piC runs which were first rescaled so their corresponding ALL runs match twentieth century observed yearly rainfall, as in (**c**).

To test the role of internal variability in the CMIP5 fully coupled models, we cannot use the MMM, because internal variability will have differing phase across different simulations. Instead, we examine power at different frequencies in individual coupled runs. Figure 5a compares the padded power spectrum (PS) of twentieth century observed Sahel precipitation (solid black) to the padded PS of the ALL simulations, first estimated for individual runs, then averaged across ensemble members for each model. They are colored by the difference in the modelled and observed rainfall climatology from 1901 to 2003, where brown is used for models which are drier than observations, grey is used for models whose climatologies are near the observed climatology, and turquoise is used for models which are wetter than observations. As the individual ALL runs are single realizations, compounding forced and internal variability like observations, they are directly comparable to observations.

While there are three models (MIROC-ESM p1, MIROC-ESM-CHEM p1, and GFDL-ESM2G p1) which nearly reach the high power of the observations at a period of 100 years, these models are biased wet, and also exhibit over-estimates of high-frequency variability. Figure 5b displays the PS of the estimate of observed internal variations implied by the MMM, calculated as the residual of observations with respect to the ALL MMM (black dashed-dotted line), and compares it to its modeled counterpart, estimated as the mean PS by model of the individual piC runs, colored by the same rainfall biases used in Fig. 5a. Since there is often only one piC simulation per model, in order to reduce uncertainty in the PS, the long piC runs are divided into continuous, non-overlapping sections, and PS are taken separately for each section and then averaged together. We again see that wet models overestimate high-frequency variability, and no model matches the low-frequency power of the residual, pointing to inconsistency between model simulations, their MMM, and observations. If the models underestimate forced variability, or if the MMM underestimates the magnitude of the modelled forced variability, this will cause the estimate of observed internal variability to be too large; so while this comparison allows us to make a statement about consistency, it does not determine whether it is simulated internal variability or our estimate of forced variability that is incorrect. However, it is clear that modelled internal variability does not contribute substantial power at low frequencies.

The PS for both the forced and piC runs are clearly stratified by modelled precipitation climatology. To investigate whether any of the models capture the observed distribution of power across different frequencies, in Fig. 5c and d we rescale the simulations by model before taking the PS and the mean by model so that the climatology of each model’s ALL simulations matches observed rainfall climatology. This mostly destroys the stratification in the previous panels (see Figure S1). The distribution of model-mean scaled ALL and piC PS are represented by blue and yellow shaded areas in Fig. 5c and d, respectively. The blue and orange dashed lines in Fig. 5c and d mark the centers of these distributions with 3-tiered, unweighted means over the PS of the ALL and piC runs, respectively. The other colored dashed lines in Fig. 5c mark the tiered means over the PS of all runs in each of the three individual forcing experiments (pink = AA, brown = NAT, green = GHG) for comparison.

The black dashed line in Fig. 5c shows the sum of the tiered mean piC PS (orange dashed line from Fig. 5d) and the PS of the ALL MMM (i.e. the blue line in Fig. 3). If the MMM accurately represented the simulated forced power when scaled to the observed climatology, we would expect this sum to match the tiered mean ALL PS (blue dashed line). Instead, it falls short at low frequency, suggesting that the ratio of the variance of the ALL MMM to observed climatology underestimates the ratios of simulated forced variance to climatological Sahelian precipitation in CMIP5 models. This may be because the ensemble is biased dry, or because differing responses to forcing between models cause the consensus forced response to have lower variance than exhibited in individual models. In addition to any implications for the RMSE calculations displayed earlier, this means that the residual spectrum in Fig. 5d is an overestimate of internal variability in observations as implied by the CMIP5 ensemble.

However, it is still clear that even scaled piC simulations do not exhibit any increase in power at low frequency (Fig. 5d). Even though the inclusion of external forcing introduces low-frequency variance (Fig. 5c), the CMIP5 models are unable to capture the scale of the increase in power at low frequency in the observed PS, which exceeds the 95th percentile of rescaled ALL PS at periods longer than 65 years. Of the different forced experiments, ALL and AA are the only ones that exhibit substantial multi-decadal variability. Thus, while the variance of the ALL MMM is somewhat underestimated, the vast majority of the discrepancy in low-frequency power between simulations and observations is not due to attenuation in the MMM, but rather to model deficiency, whether in capturing the full magnitude of the forced response to AA, or in detailing the true character and magnitude of the other forced responses, low-frequency internal variability, and their interactions.

## Discussion

The analysis in this study shows that the consensus response of Sahelian precipitation to twentieth century external forcing in CMIP5 simulations, as defined by the 3-tiered multi-model mean (MMM), correlates significantly with observations. It further shows that *both* anthropogenic aerosols (AA) and volcanic aerosols (NAT) contribute significantly and substantially to making the CMIP5 MMM similar to observations, with AA mostly responsible for the multidecadal forced variability. Given that the performance of the ALL MMM can apparently be explained with AA and NAT alone, we conclude that GHG do not contribute to the consensus forced response of Sahel seasonal precipitation in CMIP5 models. This does not mean that GHG do not influence Sahelian precipitation in any way, or that GHG will not play a significant role in the future as the magnitude of the forcing increases. While some individual models have indicated a role for GHG in the recovery since the mid 1990s^15^, it is possible that the models as an ensemble do not yet capture the effects of GHG on Sahelian rainfall because the magnitude of the forcing is still too small over the historical period. Alternatively, competition between the mechanisms linking GHG forcing to Sahelian rainfall may have masked the effects of GHG by cancelling within individual simulations^26^ or between models^34^ in the MMM. Finally, it has been suggested that the response to GHG is inherently non-linear (e.g. different circulation responses to different magnitudes of warming in Neupane and Cook^36^), or interacts non-linearly with other forcings (e.g. the interaction of an “upped ante” and changing moisture supply, as suggested by Giannini and Kaplan^16^). These non-linearities are difficult to test without the ability to compare the ALL MMM to “all but GHG” simulations, which are not widely available in CMIP5.

While the root mean squared error (RMSE) of the ALL MMM with observations is also significantly different from noise, it is 96% of the observed rainfall variance, meaning that modelled forced variability can hardly account for observed variability since the ALL MMM is hardly better than a constant prediction. Our residual consistency test showed that while the MMM is somewhat over-attenuated relative to the forced response in individual models, the discrepancy between total observed variability and total modelled variability is an order of magnitude larger, and modelled internal variability cannot account for the difference between the simulated forced response and observations.

Since modelled internal variability does not show substantial low-frequency variability while the AA MMM does, it is tempting to attribute the full magnitude of observed multi-decadal variability to AA, as many previous studies have done by focusing only on standardized trends^11^, correlations^16^, or detectability in a fingerprinting framework^18,23^. However, such a claim would rely heavily on assumed grid-point linearity of the climate response to different forcings, which is disputed for tropical rainfall (i.e. Giannini and Kaplan^16^ on GHG and anthropogenic aerosols, Polson, et al.^18^ on the indirect aerosol effect and spatial trend patterns in the Asian Monsoon, Lohmann and Feichter^33^ on feedbacks involving the indirect aerosol effects, Neupane and Cook^36^ on GHG-induced circulation changes over Africa, and Meehl, et al.^48^ on non-linear feedbacks between solar forcing and GHG), as well as on the accuracy of simulated forced and internal variability. In fact, it is not possible to say without further investigation into the physical pathways influencing Sahelian precipitation whether the model deficiency is in the modelled response to forcing or in modelled internal variability. Given the strong link between Sahelian rainfall and North Atlantic SST^13,14,16,38,49^, it is perhaps not a coincidence that models lack strong low-frequency variability both in Sahel rainfall and in internally-generated Atlantic Multidecadal Variability in SST (AMV)^50^. The community is currently still debating whether the observed AMV is forced by AA^20,22,30^ or is an internal phenomenon which is linked to ocean circulation variability^37,51–54^ and is dramatically underestimated in most models^50^.

Future work that focuses on characterizing and quantifying the mechanisms of influence on Sahelian precipitation in simulations and observations and using the next generation of climate models^55^ might shed new light on whether the model/observation discrepancy documented here is due to an underestimate in the strength of the precipitation response to AA or a failure of CMIP5-class climate models to capture low-frequency internal variability.

## Methods

### Data

Our index of Sahel rainfall variability is land-averaged precipitation anomalies for the monsoon season (July–September; JAS) over the region 12°–18°N, 20°W–40°E. For precipitation observations we use the Global Precipitation Climatology Center (GPCC) dataset^44^, which is quite similar to the Climate Research Unit (CRU)^45^ dataset in average precipitation over the Sahel. The two are compared in Fig. 1, and GPCC is used for the rest of the paper. Model simulations come from the Coupled Model Intercomparison Project phase 5 (CMIP5)^43^, which includes simulations by over 50 models from 20 different research institutions. Not all models contribute simulations to all four historical experiments; we use all available runs (between 1 and 10 for a given model) from all models (distinct name or physics number) and research institutions that have complete data from 1901 (where the observed rainfall record begins) to 2003 (where some models stop their historical simulations). There are 14 models from 8 institutions that contributed model simulations to the AA experiment, 21 models from 15 institutions that contributed to the GHG experiment, 22 models from 15 institutions that contributed to the NAT experiment, and 51 models from 20 institutions that contributed to the ALL experiment (Table S1). Here, if the physics number is changed, it is treated as a different model under the same institution.

### The multi-model mean

The MMM is defined as a 3-tiered, weighted average: (1) across individual simulations (runs) to get an ensemble mean (EM) for each model, (2) across EMs to get an institution mean (IM) for each research institution, and (3) across IMs to get the multi-model mean (MMM) for that experiment. While any averaging helps to filter internal variability from the MMM, the first tier focuses on reducing internal variability present in the individual runs, the second tier focuses on reducing variability between models from uncertainty in parameter values, and the third tier focuses on reducing variability between institutions from uncertainty in parameterization. A simple mean across all model simulations is very similar to the tiered mean (not shown), but tiers are used to prevent over-representation of particular parameterizations and parameter choices in the MMM and in the uncertainty and significance calculations (which will be described below under “Uncertainty and significance: bootstrapping and randomized bootstrapping”).

If a random variable (such as the internal variability component of yearly JAS Sahel precipitation) has a variance of $\sigma^{2}$, then the mean over *n* realizations of that variable will have a variance of $\sigma^{2} /n$. The forced variability component may experience some attenuation as well due to differences in the simulated response to forcing between models. Given that the forced signal ought to be similar across simulations, we expect attenuation of internal variability to overwhelm attenuation in forced variability. Thus, means over models with more runs or over institutions with more models will have a higher signal (forced variability) to noise (internal variability) ratio than their counterparts. However, they will also have less total variability, causing them to (counterproductively) contribute *less* to the MMM than their more noisy counterparts. We counteract this by using weights which are inversely proportional to the expected attenuation of noise in the MMM tiers.

For a weighted mean $\sum\nolimits_{i} {w_{i} X_{i} }$ between independent random variables *X*_*i*_ with mean $\mu_{i}$, variance $a_{i} \sigma^{2}$, and weight *w*_*i*_, where $\sum\nolimits_{i} {w_{i} = 1}$, we find that:

$$ \sigma_{{\sum\nolimits_{i} {w_{i} X_{i} } }}^{2} = {\text{E}}\left[ {\left( {\mathop \sum \limits_{i} w_{i} X_{i} } \right)^{2} } \right] - {\text{E}}\left[ {\mathop \sum \limits_{i} w_{i} X_{i} } \right]^{2} = \mathop \sum \limits_{i} w_{i}^{2} \left( {{\text{E}}\left[ {X_{i}^{2} } \right] - \mu_{i}^{2} } \right) = \sigma^{2} \mathop \sum \limits_{i} w_{i}^{2} a_{i} $$

Thus, to counteract the attenuation from a previous tier, captured in *a*_*i*_, we define the weights as $w_{i} = a_{i}^{ - .5} /\sum\nolimits_{i} {a_{i}^{ - .5} \propto a_{i}^{ - .5} }$. Specifically, let *f*, *i*, *m*, *r*, *N*_*f*_, *N*_*fi*_, and *N*_*fim*_ be such that each forcing experiment *f* is simulated by *N*_*f*_ institutions, with *N*_*fi*_ models from each institution *i*, and *N*_*fim*_ runs from each model *m*, and assume that the JAS Sahel precipitation in a given year for each run *r* has a variance of $\sigma^{2}$. In the first tier, where $a_{fimr} = 1$ and $w_{fimr} = \frac{1}{{N_{fim} }}$ (an unweighted mean), we find that the variances of the EMs are $\sigma_{{EM_{fim} }}^{2} = \sigma^{2} \sum\nolimits_{r} {\frac{1}{{N_{fim}^{2} }} = \frac{{\sigma^{2} }}{{N_{fim} }}}$, giving $a_{fim} = \frac{1}{{N_{fim} }}$ for the second tier. To combat this attenuation, in the second tier we define weights $w_{fim} = \frac{{\sqrt {N_{fim} } }}{{\mathop \sum \nolimits_{m} \sqrt {N_{fim} } }} = \frac{{\sqrt {N_{fim} } }}{{M_{fi} }} \propto \sqrt {N_{fim} }$, where $M_{fi} = \sum\nolimits_{m} {\sqrt {N_{fim} } }$ is the normalization constant for those weights. Using these weights, the variances of the IMs are $\sigma_{{IM_{fi} }}^{2} = \sigma^{2} \sum\nolimits_{m} {\frac{{N_{fim} }}{{M_{fi}^{2} }} \frac{1}{{N_{fim} }} = \frac{{N_{fi} }}{{M_{fi}^{2} }}\sigma^{2} }$, giving $a_{fi} = \frac{{N_{fi} }}{{M_{fi}^{2} }}$ for the third tier. Then in the third tier, $w_{fi} \propto \frac{{M_{fi} }}{{\sqrt {N_{fi} } }}$.

### Approach

MMMs are compared to observations using correlations, which capture similarity in frequency and phase, and root mean squared errors (RMSE), which capture differences in magnitude and are expressed as a fraction of observed variance. When comparing the observations to themselves, the correlation would be 1 and the RMSE would be 0; when comparing the observations to a constant prediction, the correlation would be 0 and the RMSE would be 1 (or 100% of observed variance).

### Uncertainty and significance: bootstrapping and randomized bootstrapping

Estimates of sampling uncertainty over all possible model parameterizations are obtained by bootstrapping (resampling with replacement) available forced IMs before calculating the MMM and corresponding correlations and RMSE, yielding probability density functions (PDF) around the MMM correlation and RMSE. This PDF can also be interpreted as a measure of agreement between CMIP5 models.

To test the null hypothesis—that all results from the forced experiments are consistent with noise in the MMM derived from modelled internal variability alone—we measure uncertainty in the MMM by repeating the bootstrapping procedure once for each of the four forced experiments, using the long, constant-forcing preindustrial control (piC) runs from the set of models contributing historical simulations to that experiment, choosing random, continuous, 103-year subsets before each bootstrap (referred to as “randomized bootstrapping”).

In addition to uncertainty derived from model parameterization, the MMM still contains noise from lingering coincident internal variability, and because bootstrapping underestimates variance when sample size is small, this procedure does not capture the full magnitude of that uncertainty (when randomizing is not used while boostrapping the piC MMMs, for comparison, the piC confidence interval contains high-frequency variability similar to that seen around the forced MMMs in Fig. 2, not pictured). However, the length of the piC runs allows us to effectively increase the sample size of 103-year runs in the randomized bootstrapping method enough to give an accurate estimate of noise uncertainty in the MMM: this is evident from the nearly-uniform confidence intervals of the piC MMMs (yellow, Fig. 2), which contain no time-varying signal by construction.

### Residual consistency

We evaluate consistency between modelled and observed internal and externally-forced variability by examining and comparing the power spectra (PS) of individual ALL and piC simulations. For increased sampling of frequencies we zero-pad the time series before taking the PS, and for clarity and decreased uncertainty, we average across PS from the same model before presenting the PS. This is less effective for the piC simulations, which usually contain only one (long) simulation per model. To help reduce uncertainty in the piC PS, we divide them into consecutive, non-overlapping segments of 103 years, calculate the PS of the segments separately, and average them together. To calculate the rescaled PS, we scale the individual ALL and piC runs from a given model by (mean observed twentieth century precipitation)/(mean precipitation from the ALL runs for that model) before taking the PS. We then average the PS by model and present the 66% and 95% range of the PS. We also present 3-tiered, unweighted means over all simulations of the rescaled ALL, AA, NAT, and GHG PS. The mean PS are calculated using an unweighted mean because different realizations of internal variability in the simulations do not cause attenuation of the spectral peaks characterizing that variability.

## Supplementary information

Supplementary information.

## Data Availability

Global Precipitation Climatology Center (GPCC)^44^ observational data is freely available online (see https://www.esrl.noaa.gov/psd/data/gridded/data.gpcc.html) and CMIP5^43^ model data is freely available through the Earth System Grid (see https://esgf-node.llnl.gov/projects/esgf-llnl/).
