# Supplementary material for: The effects of anthropogenic and volcanic aerosols and greenhouse gases on twentieth century Sahel precipitation
Source: Sci Rep. 2020 Jul 22;10:12203. doi: 10.1038/s41598-020-68356-w (PMC7376254; doi:10.1038/s41598-020-68356-w)
Supplement: Supplementary file 1 — Supplementary information. [file 41598_2020_68356_MOESM1_ESM.pdf]

Supplementary Information for

The effects of anthropogenic and volcanic aerosols and greenhouse gases on twentieth century Sahel precipitation

Rebecca Jean Herman[1]*, Alessandra Giannini[23], Michela Biasutti[4], and Yochanan Kushnir[4]

[1]*Department of Earth and Environmental Sciences, Columbia University, New York, NY, USA*

[2]*International Research Institute for Climate and Society*
The Earth Institute at Columbia University, New York, NY, USA

[3]*Laboratoire de Météorologie Dynamique/IPSL,*
École Normale Supérieure, PSL Research University,
Sorbonne Université, École Polytechnique, IP Paris,
CNRS, Paris, France

[4]*Lamont-Doherty Earth Observatory of Columbia University, Palisades, NY, USA*

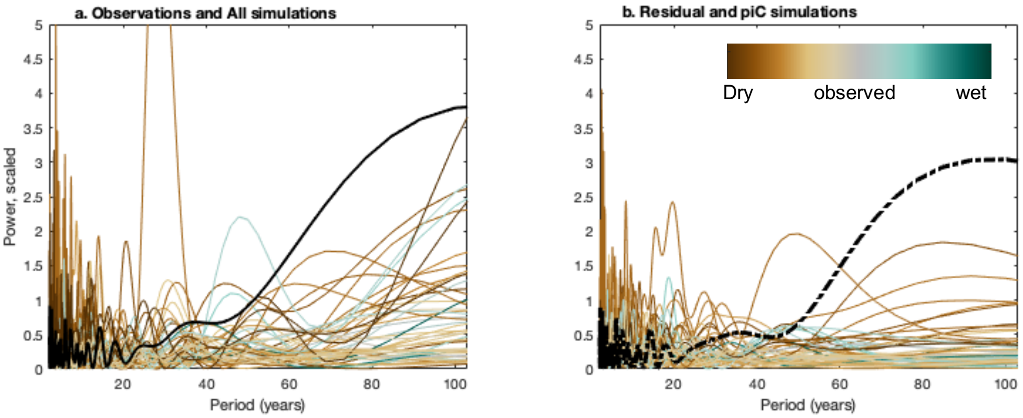

Figure S1. Scaled Stratification: Same as Figures 5c and 5d but displayed as in Figures 5a and 5b.

Power spectra (PS) of observed 20th century Sahel rainfall (solid black, a) and the residual after removing the ALL MMM (black dotted-dashed, b), and mean PS by model of individual ALL (a) and piC (b) runs which were first rescaled by model so their corresponding ALL runs match 20th century observed JAS rainfall, colored by original simulated average JAS rainfall bias of the ALL runs compared to 20th century observations, where observed rainfall is grey, wet models are turquoise, and dry models are brown. piC PS are averaged over multiple segments of the simulations.

Figure S1 shows the scaled power spectra (PS) from Figures 5c and 5d in the style of Figures 5a and 5b, where the PS for each model (averaged over the runs for that model, and in the case of the piC, over different sections of the long piC run) are represented separately, colored by the rainfall bias of that model's ALL runs relative to observations. While the correction seems to

completely get rid of the stratification by total rainfall bias at medium and low frequency in the ALL simulations, it seems to overcorrect the power in the simulations of the driest models at high frequency in the ALL simulations, and at all frequencies in the piC simulations. This is perhaps not surprising, as when a model is particularly dry, normal variability may make up a larger fraction of the total rainfall. As this correction is imperfect, we do not use it in the calculation of the MMM; rather, only to facilitate comparison of the models in Figure 5.

Table S1 displays the models and runs used in this study, as well as their institution classifications.

Models	ALL				AA				GHG				NAT	
	p	Num runs used	runs excluded	reason	p	Num runs used	runs excluded	reason	p	Num runs used	runs excluded	reason	p	Num runs used
ACCESS1-0	1	1												
ACCESS1-3	1	1							1	1			1	3
bcc-csm1-1	1	3							1	1			1	1
bcc-csm1-1-m	1	3												
BNU-ESM	1	1							1	1			1	1
CanCM4*	1		all	no data before 1961										
CanESM2	1	5			4	5			1	5			1	5
CCSM4	1	6			10	3			1	3			1	4
					14									
CESM1-BGC	1	1				2	r6i1p14	access error						
CESM1-CAM5	1	3			10	3			1	1	r1i1p1, r2i1p1	contain NaN	1	3
CESM1-CAM5-1-FV2*	1	4												
CESM1-FASTCHEM	1	3												
CESM1-WACCM	1	1	r4i1p1, r3i1p1, r2i1p1	no data before 1955										
CMCC-CESM	1	1												
CMCC-CM	1	1												
CMCC-CMS	1	1												
CNRM-CM5	1	10							1	6			1	6
CNRM-CM5-2	1	1												
CSIRO-Mk3-6-0	1	10			4	5			1	5			1	5
EC-EARTH	1	1												
FGOALS-g2	1	4	r2i1p1	no data before 1902	1	1			1	1			1	3
FGOALS-s2	1	3												
FIO-ESM	1	3												
GFDL-CM3	1	5			1	3			1	3			1	3
GFDL-ESM2G	1	3												
GFDL-ESM2M	1	1			5	1			1	1			1	1
GISS-E2-H	1	6			107	5			1	5			3	5
	2	5			310	5							1	5
GISS-E2-H-CC	1	1												
GISS-E2-R	1	6			107	5			1	5			3	5
	2	5			310	5							1	5
	3	5												
GISS-E2-R-CC	1	1												
HadCM3*	1	10												
HadGEM2-AO	1	1												
HadGEM2-CC	1	1	r3i1p1, r2i1p1	no data before 1960										
HadGEM2-ES	1	4							1	4			1	4
inmcm4	1	1												
IPSL-CM5A-LR	1	6			3	1			1	3			1	3
IPSL-CM5A-MR	1	3							2	3				
IPSL-CM5B-LR	1	1												
MIROC-ESM	1	3							1	3			1	3
MIROC-ESM-CHEM	1	1							1	1			1	1
MIROC4h	1		all	no data before 1950										
MIROC5	1	5												
MPI-ESM-LR	1	3												
MPI-ESM-MR	1	3												
MPI-ESM-P	1	2												
MRI-CGCM3	1	3							1	1			1	1
	2	2												
MRI-ESM1*	1	1												
NorESM1-M	1	3			1	1			1	1			1	1
NorESM1-ME	1	1												
Total Models used	51				14				21				22	

*Table S1. Models and runs used in this paper for the different forcing experiments. "p" is the physics number – different physics numbers within the same model are treated as different models. Blank spaces exist in the chart where there were no runs from that model under that forcing experiment. *no accompanying piC run. Doubled lines divide different research institutions.*
